# Case Report: A Case of Concomitant Paroxysmal Kinesigenic Dyskinesia and Epilepsy: Can We Treat Two Birds With One Stone?

**DOI:** 10.3389/fneur.2022.826897

**Published:** 2022-02-02

**Authors:** Jun-Hong Geng, Yang Zheng, Quan-Fu Li, Qun Hou, Xiao-Hang Wang, Yan Jiang

**Affiliations:** Department of Neurology, The First Affiliated Hospital of Zhejiang Chinese Medical University, Hangzhou, China

**Keywords:** lacosamide, paroxysmal kinesigenic dyskinesia, epilepsy, movement disorder, PRRT2

## Abstract

**Background:**

Paroxysmal kinesigenic dyskinesia (PKD) is characterized by recurrent episodes of movement-induced motor attacks. PKD patients may have concomitant epilepsy. Differentiation between the two disorders and effective control of both diseases remain challenging.

**Case Presentation:**

We present a Chinese girl with typical manifestations of PKD, who also suffered from generalized tonic-clonic seizure attacks at the same time. Genetic testing confirmed a *PRRT2* mutation (c.649dupC). Oxcarbazepine was initially used, but withdrawn due to a hypersensitivity reaction. Levetiracetam was initiated afterwards, which was effective for seizures but failed to control her PKD symptoms. The addition of lacosamide (LCM) completely controlled her PKD symptoms.

**Conclusion:**

This is the first case reporting the effectiveness of LCM for concomitant PKD and epilepsy. Our case proposes a novel alternative for such patients who are resistant or cannot tolerate conventional anti-sodium antiepileptics.

## Introduction

Paroxysmal Kinesigenic Dyskinesia (PKD) is a rare disease characterized by recurrent, sudden attacks of dystonia, chorea, ballistic, and athetoid involuntary movements, which are triggered by sudden voluntary movements (1). The diagnosis of PKD is mainly based on features including movement-induced attacks with a short duration, reserved consciousness, a dramatic antiepileptic drug (AED) responsiveness and a positive family history or proline-rich transmembrane protein 2 (*PRRT2*) mutation (2). It has been reported that 8% of PKD had concomitant epilepsy (3). Despite its rarity, patients with concomitant PKD and epilepsy remain the biggest challenge, since a correct diagnosis remain difficult and a proportion were reported to be resistant to AEDs treatment (4, 5).

The first challenge in managing patients with both PKD and epilepsy lie in the diagnosis, given the similar presentations as paroxysmal recurrent motor attacks. Two-thirds of patients with PKD was misdiagnosed with epilepsy (3). The other challenge is treatment. Effective drugs remain unknown for patients with both PKD and epilepsy. Previous reports mostly use a trial-and-error strategy when choosing drugs, which might bring extra stress and economic burdens.

In this study, we described a 15-year-old Chinese girl, who carried a *PRRT2* gene mutation, presenting with concomitant PKD and epilepsy. Her movement symptoms had an excellent response to LCM. We also summarized previous reports on concomitant PKD and epilepsy, hoping to address the diagnostic and therapeutic challenges in such patients.

## Case Report

The 15-year-old girl was born at 38 weeks of gestation by vaginal delivery to a pair of non-consanguineous Chinese parents. She was the only child and her prenatal period was uneventful. Her growth and developmental milestones were normal.

At age of 5, she suffered from a seizure at sleep in the early morning, characterized by limb twitching and jerking, with loss of consciousness, which lasted for 3 min. She complained of weakness and headache after the attack. She was subsequently sent to the hospital where the electroencephalogram (EEG) and magnetic resonance imaging (MRI) were both non-revealing. At the age of 8, she presented with paroxysms of abnormal involuntary posturing of both hands, ranging from a few seconds to 1 min. The episodes were mostly triggered by sudden movement or emotional stress. Her consciousness was fully preserved during the attacks. Her family history was negative for seizures. However, her mother had dyskinesia after sudden movements when she was young, such as suddenly standing up or running. Her mother's symptoms completely disappeared after the age of 16. Additionally, her mother's younger sister and cousin had similar dyskinesia.

The girl went to a tertiary hospital and was diagnosed with PKD at the age of 10. She was initially treated with oxcarbazepine, which unfortunately, caused an allergic reaction in the form of skin rashes. Levetiracetam was initiated, with an initial dose of 500 mg twice daily. However, she still had paroxysmal dystonia despite the levetiracetam treatment, with a frequency of about once per week.

**Figure 1:**
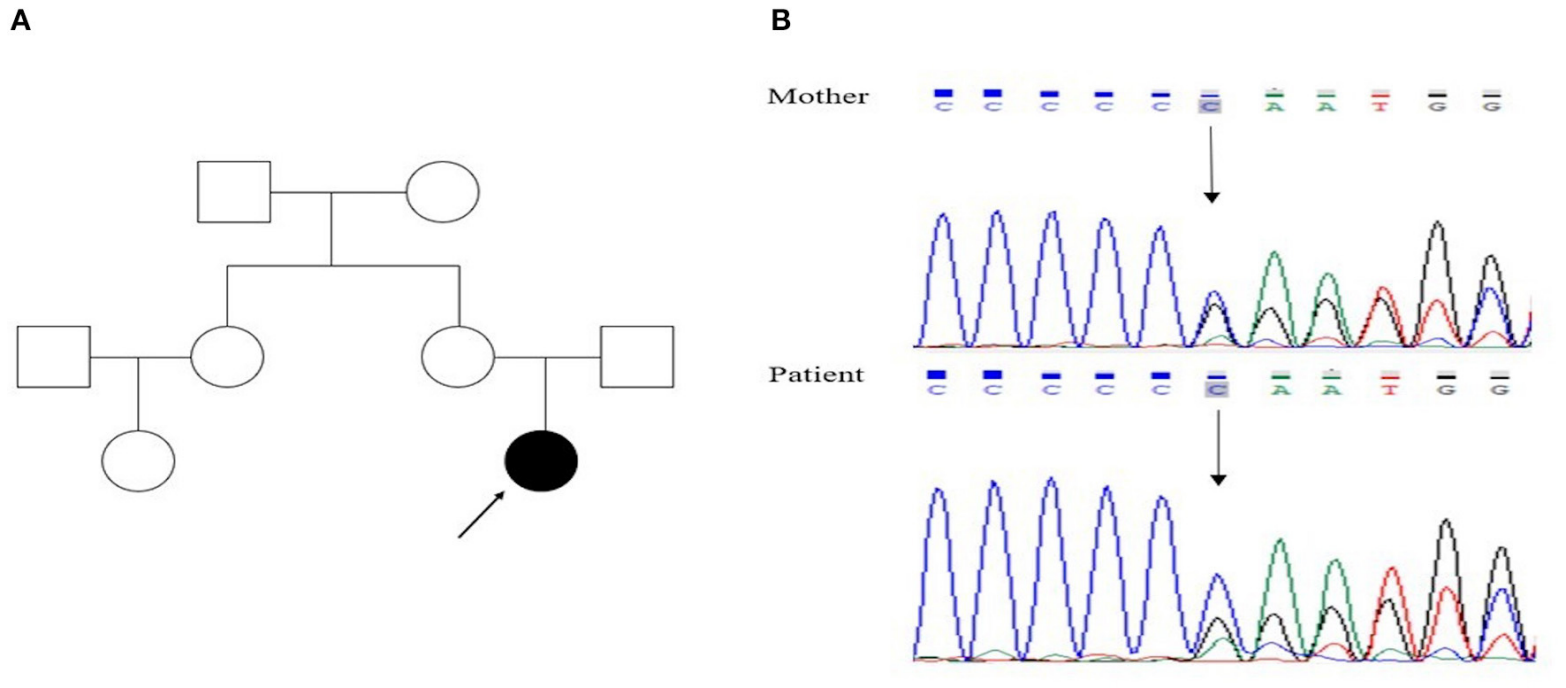
**(A)** Family pedigree of the proband (filled symbols represent the patient, open symbols normal persons, index case indicated by arrow); **(B)** genetic analysis showed that both the patient and her mother had a heterozygous mutation, c.649dupC, in the *PRRT2* gene.

**Figure 2:**
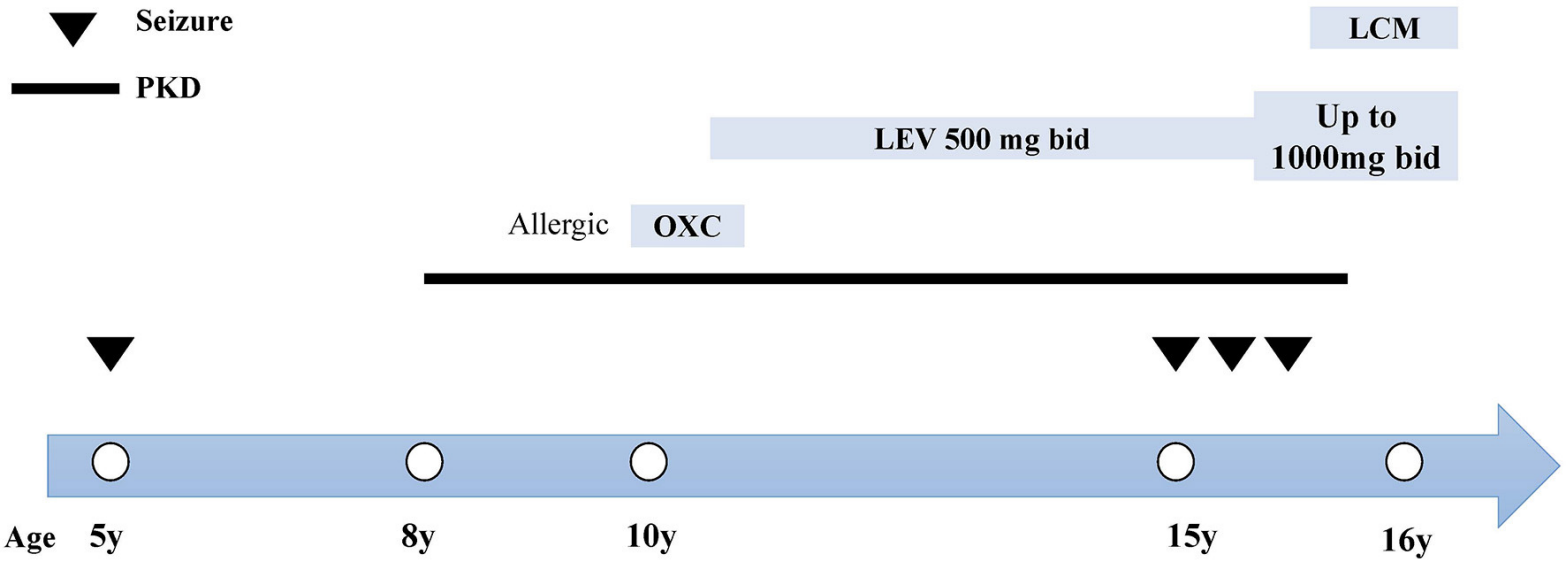
Clinical course and treatment of the case. The x-axis indicates the age of patient at each attack. Epileptic seizures are indicated by inverted triangles. The course of PKD is denoted by the black line. The name, dosage and timing of drug treatment are shown at the top of the diagram. bid, twice daily; LCM, lacosamide; LEV, levetiracetam; OXC, oxcarbazepine.

At the age of 15, she suffered from another seizure at sleep in the early morning, characterized by limb twitching and jerking with loss of consciousness, which lasted for around 3 min. Weakness and headache were reported after the attack. EEG at an outside institute showed the presence of scattered theta waves, sharp waves and sharp slow waves induced by hyperventilation. Long-term EEG showed intermittent bilateral frontal slowing. Neurologic examinations and brain MRI were normal at that time. Similarly, she experienced two grand mal seizures in the next month, both at sleep in the early morning. Dosage of levetiracetam was increased to 1,000 mg twice daily. However, the paroxysmal dystonia persisted. The diagnosis of concomitant PKD and epilepsy was considered. Genetic testing revealed a heterozygous frameshift mutation of c.649dupC (p.Arg217Profs^*^8) (Figure 1). LCM, at a dose of 50 mg twice daily, was added to the previous treatment. The patient responded excellently to the treatment. Neither dystonia nor seizures recurred after the LCM treatment (Figure 2). No adverse effects such as cardiac abnormalities and mental disorders were observed. The patient remained free of both PKD and seizures at the last follow-up 10 months after the initiation of LCM.

## Discussion

Herein, we report a case of an adolescent female who had concomitant PKD and epilepsy with a *PRRT2* mutation (c.649dupC). The diagnostic pearls for patients with episodic motor attacks are listed. We also discuss managements for patients with concomitant PKD and epilepsy. The excellent efficacy of even low-dose LCM highlights a new therapeutic option for such patients.

The first challenge in our case lies in the correct diagnosis of PKD and epilepsy. Both disorders can present with recurrent movements and both have a good initial response to anti-sodium-channel AEDs, though epilepsy patients would develop drug resistance later in disease course (6). To differentiate PKD and epilepsy, a careful history taking is vital. Certain ancillary testing including EEG, MRI and genetic testing would be helpful as well. PKD has an age of onset between 1 and 20 years of age with attacks consistently triggered by sudden movements, sometimes in the context of emotional, or physiologic stress (7). Kinesigenic paroxysmal movements in PKD are short and frequent. The patient has a reserved consciousness during attacks. Neurological examination and EEG are always normal (1). Epileptic seizures can mimic PKD as well, though seizure attacks are highly stereotyped and may occur during sleep. Mutations in *SCN8A, CHRNA4, KCNT1*, and *DEPDC5* were associated with epilepsy (8, 9), while *PRRT2* mutation was frequently related to PKD. In this patient, the movement-induced motor attacks are diagnosed with PKD, given the presence of triggers like sudden movements or emotional stress, the normal results on repeated MRI, a positive family history and the presence of *PRRT2* mutation. The recurrent generalized tonic-clonic attacks all at night and presence of post-attack headache pointed toward the diagnosis of epilepsy. However, we did not further investigate the etiology of epilepsy considering the financial burdens of the patient. Herein, given a thorough history taking and evidence from EEG and genetic evidence, we diagnose the patient with concomitant PKD and epilepsy.

PKD is characterized by an exquisite response to antiepileptic drugs. First line treatments are carbamazepine, oxcarbazepine and phenytoin (1, 10). A dramatic response to antiepileptic drugs is seen in 98.4% of *PRRT2*-PKD patients (7). Treatment failure was mainly reported in homozygous or compound heterozygous *PRRT2* mutation carriers (11, 12). Importantly, effective treatments to control both PKD and epilepsy remain undetermined, as shown in Table 1 (3–5, 13–19). Allergic reactions to certain AEDs like carbamazepine or oxcarbazepine further limited their use in such patients. Therapeutic options, both safe and efficacious, are urgently needed for those with concomitant PKD and epilepsy.

**Table 1:** Clinical characteristics of previously-reported patients with concomitant PKD and epilepsy.

**References**	**Sex**	**Age at onset of epilepsy**	**Age at onset of PKD**	**Symptomatology**	**Family history** **of PKD**	**Brain CT or MRI**	**Scalp EEG**	**Causative gene**	**Ineffective drugs**	**Effective drugs**
Whitty et al. (4) Hudgins et al. (13) Jung et al. (14) Tan et al. (3) Guerrini et al. (5) Cuenca et al. (15) Prashantha et al. (16) Seo SY et al. (17) Tanabe Y et al. (18) Zhang et al. (19)	Male Female Male Female Male Male Male Female Female Male Male Female Female Male	12y 13y 5y 11y 8y 12y 16y 2y6m 4m – 23y 7y – 8y	13y Infancy 8m 2y 3y 12y 4.5y 3m – – 18y 6m 14y 9y	GTCS GTCS GTCS GTCS GTCS GTCS GTCS Absence GTCS GTCS GTCS Hypotonic GTCS GTCS	No – Yes Yes Yes Yes No No Yes Yes No Yes No No	– – – – – Normal – Normal Normal Normal Calcifications Normal Normal Calcifications	Sharp wave Normal Normal Normal Normal Normal Spike-and-wave Spike-and-wave Normal Normal Normal CTS Slow wave Sharp wave, sharp slow wave	– – – – – – – – – – – *PRRT2* *PRRT2* –	PMD+ PB – – – – – – VPA, CBZ, BZDs, LTG+TPM – – PHT – CZP, LEV –	PMD+PHT MPB+PHT MPB+PHT MPB+PHT PHT+PB PHT PB LTG+ESM PB CBZ Ca, Vit D, CBZ OXC CBZ Ca, Vit D, CBZ

*GTCS, generalized tonic-clonic seizures; PMD, primidone; PB, phenobarbital; PHT, phenytoin; MPB, mephobarbital; VPA, valproate; CBZ, carbamazepine; BZDs, benzodiazepines; LTG, lamotrigine; TPM, topiramate; ESM, ethosuximide; OXC, oxcarbazepine; CZP, clonazepam; Ca, calcium; Vit D, vitamin D; LEV, levetiracetam; CTS, centrotemporal spike*.

LCM is a third-generation antiepileptic drug, with unique mechanisms of enhancing the slow inactivation of voltage-gated sodium channels. Unlike other traditional sodium channel blockers such as phenytoin and carbamazepine which inhibit the fast inactivation of the channels, LCM mainly reduces the long-term availability of sodium channels for activation, resulting in the normalization of activation thresholds (20). Mathew et al. reported a 19-year-old boy with PKD, who was responsive to LCM treatment (at a dose of 50 mg twice daily) after a hypersensitivity reaction of oxcarbazepine (21). Another recent retrospective study included four children with PKD, one of them had a *PRRT2* mutation (c.649dupC). The low dose of LCM showed an excellent efficacy in all children, regardless of the status of *PRRT2* mutations (22). However, large sampled studies on the correlation between *PRRT2* mutations and LCM response is lacking. Our patient with concomitant *PRRT2*-positive PKD and epilepsy had a good response to even low-dose LCM after an allergic reaction to oxcarbazepine and treatment failure with levetiracetam. Our case further demonstrated the efficacy of low-dose LCM in patients with concomitant PKD and epilepsy, offering an optimal and safe therapeutic option for such patients.

To our knowledge, this is the first case using LCM for concomitant *PRRT2* PKD and epilepsy. We suggest that the use of low-dose LCM might be an efficacious, safe and tolerable option for such patients. Further studies with a large cohort are needed to confirm our findings, especially those who are allergic or unresponsive to carbamazepine and oxcarbazepine.

## Data Availability Statement

The original contributions presented in the study are included in the article/supplementary files, further inquiries can be directed to the corresponding author.

## Ethics Statement

The studies involving human participants were reviewed and approved by Ethics Committee of the First Affiliated Hospital of Zhejiang Chinese Medical University. Written informed consent to participate in this study was provided by the participants' legal guardian/next of kin.

## Author Contributions

J-HG drafting of the article, clinical examination of the patient, and bibliographic search. YZ and YJ conceptualized and designed the study, coordinated and supervised data collection and analyzation, and critically reviewed and revised the manuscript. Q-FL, QH, and X-HW contributed to conception and design of the study. All authors contributed to the article and approved the submitted version.

## Funding

This work was supported by the Natural Science Foundation of Zhejiang Province (CN) under Grant No. LY19H090001 and the Health Science and Technology Project of Zhejiang Province (CN) under Grant No. 2022KY228.

## Conflict of Interest

The authors declare that the research was conducted in the absence of any commercial or financial relationships that could be construed as a potential conflict of interest.

## Publisher's Note

All claims expressed in this article are solely those of the authors and do not necessarily represent those of their affiliated organizations, or those of the publisher, the editors and the reviewers. Any product that may be evaluated in this article, or claim that may be made by its manufacturer, is not guaranteed or endorsed by the publisher.
